# Feature selection using distributions of orthogonal PLS regression vectors in spectral data

**DOI:** 10.1186/s13040-021-00240-3

**Published:** 2021-01-22

**Authors:** Geonseok Lee, Kichun Lee

**Affiliations:** Industrial Engineering, Hanyang University, Seoul, Korea

**Keywords:** Feature selection, PLS, Orthogonal signal correction, Regression vector, Permutation test

## Abstract

Feature selection, which is important for successful analysis of chemometric data, aims to produce parsimonious and predictive models. Partial least squares (PLS) regression is one of the main methods in chemometrics for analyzing multivariate data with input *X* and response *Y* by modeling the covariance structure in the *X* and *Y* spaces. Recently, orthogonal projections to latent structures (OPLS) has been widely used in processing multivariate data because OPLS improves the interpretability of PLS models by removing systematic variation in the *X* space not correlated to *Y*. The purpose of this paper is to present a feature selection method of multivariate data through orthogonal PLS regression (OPLSR), which combines orthogonal signal correction with PLS. The presented method generates empirical distributions of features effects upon *Y* in OPLSR vectors via permutation tests and examines the significance of the effects of the input features on *Y*. We show the performance of the proposed method using a simulation study in which a three-layer network structure exists in compared with the false discovery rate method. To demonstrate this method, we apply it to both real-life NIR spectra data and mass spectrometry data.

## Introduction

Feature selection is a technique to select a subset of variables which are useful in predicting target responses. From a machine learning viewpoint, irrelevant features in a prediction model deteriorate its generalization ability, and it is critical to remove such redundant features to keep the model from being misled by inappropriate information. The task of feature selection, one of the central tasks in machine learning, helps to reduce overfitting by eliminating redundant features. The prevalence of high-dimensional data becomes a challenge for researchers to perform feature selection [1]. In biological fields, particulary, high dimensionality often arises, and irrelevant and redundant features make up a high proportion of the total data [2].

Indeed, contemporary analytic methods such as near-infrared (NIR), proton nuclear magnetic resonance (^1^H NMR) spectroscopy, liquid chromatography-mass spectrometry (LC-MS), and gas chromatography-mass spectrometry (GC-MS) provide high-dimensional data sets in which the number of features is usually larger than the number of observations. Those spectral data sets, denoted by the input variables (feature) *X*, are an effective alternative to using classical chemical analyses in screening experiments [3]. They can reveal the underlying patterns associated with health characteristics, the so-called phenotypes. These include pathological characteristics, denoted by the response variables *Y*, and thus can be of substantial value in biomedical research [4]. To this end, reliable identification of features associated with response characteristics is important.

Uncovering hidden patterns associated with the response variables in spectral data sets is not trivial. One of the major problems in addressing this issue is how to deal with the existence of spectral collinearity. Spectral collinearity, originating from linear dependence among the input variables *X*, poses ill-conditioned linear equation systems. Thus, standard regression methods cannot be applied in this context, so different strategies need to be adopted. The methods usually employed for solving such problems are artificial neural networks, k-nearest neighbors, principle component analysis (PCA), and partial least squares (or projections to latent structures, PLS) among others. A detailed discussion of multivariate statistical methods in spectral data sets can be found in Holmes and Antti [5] and Lindon et al. [6].

Among these, unsupervised PCA and supervised PLS together with regression have been widely applied. These methods are useful for reducing the complexity in the feature space, the main idea is to find a low dimensional representation while retaining as much of the variation as possible. Compared to other methods, they are not only easy to interpret but also effective in explaining the interrelationships among spectral data by examining the variance levels of spectral data while being less computationally demanding. Basically, they produce a few transformed scores (positions for new directions) for the original input variables *X* to reduce the complexity of such data and retain most of the variational information.

For the task of identifying significant variables, however, produced scores by PCA often undergo lack of discrimination power since it only focuses on new directions or loadings, accounting for the maximum variation of *X*, and then projects the original input variables onto the new directions [7]. On the other hand, PLS is a type of regression model used to find the relationships between the response variables and input variables based on the assumption that they are generated by a common set of underlying factors [8]. That is to say, it finds the directions in the space of *X* that explains the maximum of variation of the space *Y*. By reducing the collinearity between input variables and increasing covariance between input and response variables at the same time, feature selection by PLS can result in a more parsimonious model without losing its predictive ability.

The task of identifying significant features via PLS faces a few challenges. The selection of original spectral variables from those transformed scores is nontrivial because they are represented as linear combinations of a large number of the original input variables. PLS loadings express the weights for these linear combinations and generate PLS regression vectors with estimated regression coefficients. The uncertainty in the estimates of PLS loadings and scores in conjunction with PLS regression vectors complicates this task. Since error in a few original input variables are propagated in all transformed scores by way of loadings, the uncertainty in PLS regression vectors increases accordingly [9, 10]. Additionally, the absence of closed-form distributions of PLS loadings also makes the task challenging. To cope with these challenges in PLS-based variable selection, several statistical approaches have been suggested. Heise applied cross-validation with a strategy of expanding neighboring variables [11]. Høskuldsson employed the stepwise regression approach based on the goodness of fit criterion [12]. Faber proposed a resampling technique [13], focusing on variables interval selection. These approaches, though known to be unbiased [14], inevitably suffer from uncertainty under a substantial number of features and often lead to the selection of unnecessary features. The presence of unnecessary features, causing overfitting, is a critical issue especially when the number of observations is less than that of features. It would be a benefit to apply a resampling method, able to regulate the selection of unnecessary features, to the identification of significant features in PLS.

This paper introduces a useful combined approach of applying orthogonal signal correction (OSC) and permutation tests to PLS for the purpose of feature selection. OSC, introduced by Wold et al. [15], removes from input variables only the part unrelated to the response variable. Extensions of OSC aiming to improve model prediction and interpretability were also presented [16, 17], sharing the same spirit in that *X* variation unrelated to the response is filtered out as a preprocessing step. Thus in the combined approach, first, OSC as a preprocessing step of PLS corrects *X* by removing systematic variation in the *X* space not correlated to *Y*. Second, we relate the orthogonal-signal corrected *X* to the PLS regression models, which we call OPLS models, and obtain OPLS-induced regression vectors. The regression vectors in OPLS models describe each variable’s contribution to the response *Y* while reflecting variables collectively on new directions. Lastly, by employing a permutation testing procedure, in which permutations of the input observations for a collection of randomly chosen features occurs, we test the significance of each coefficient in the regression vectors in a reasonably fast manner. The permutation test procedure yields empirical null distributions of weights for each individual variable’s effect on the response *Y*. The contribution of this paper is the integration of permutation test into feature selection of OPLS models combining the concepts of OSC and PLS. It then introduces the use and testing of OPLSR vectors for the purpose of feature selection, particularly in the domains of spectral data.

This paper is organized as follows. “Proposed method” section describes the combined method of orthogonal signal correction and PLS, manifesting the adopted permutation test procedure. It also includes a simulation study in which simulated data sets with a three-level network structure are used and the performance of the proposed approach is demonstrated. The proposed approach is compared with two methods: one is the Lasso, penalized linear model, and the other is the false discovery rate (FDR) method which is based on the variables’ individual effects on the response *Y* and is widely used in spectral data analysis [18, 19]. “Experiments” section demonstrates the approach with a real-life NIR data set. Finally, “Conclusions” section concludes the paper.

## Proposed method

We describe orthogonal signal correction and PLS to obtain OPLS regression vectors. Then we introduce a permutation-based test procedure to test the significance of each variable’s effect in OPLS regression vectors.

### Orthogonal PLS regression vector

In the analysis of chemometric data using a multivariate regression model *Y*=*XB*+*E*, it is common that the first component accounting for the highest percentage of the input *X* variation constitutes only a low percentage of the response *Y* variation. The input *X* and response *Y* are assumed to be mean-centered and properly normalized. In PCA, a principal component vector (or score vector) **t** is calculated by a linear combination of an input observation **x**_*j*_,*j*=1,…,*n* (row of *X*) and a loading vector **p**. Score vector **t** can be regarded as a transformed variable by the corresponding loading vector. Loading vector **p** is numerically found as an eigenvector of the sample covariance matrix *X*^*T*^*X*/(*n*−1). PLS regression, however, focuses on the covariance structure between *X* and *Y*. In PLS regression, score vectors and loading vectors for *X* and *Y* are jointly and numerically obtained to maximize the covariance between *X* score vectors and *Y* score vectors [20].

The goal of orthogonal signal correction (OSC) is to remove one or more directions in *X* (*n*×*p*) that are unrelated, or more precisely orthogonal, to *Y* and to account for the largest variation in *X* as well. OSC often serves as a pre-processing step to improve the multivariate regression model [21]. Particularly, the PLS regression coefficients after OSC have stronger interpretability and clarity. In this paper, we chose direct orthogonal signal correction since it not only bears close relations with other OSC methods and works quite well with empirical data, but also uses only least squares methods without iterative computation [22]; this means it can be analytically connected with subsequent PLS. We summarize the basic steps as follows and link them to PLS to obtain orthogonal-signal-corrected PLS regression (OPLSR) vectors that will be used for feature selection.

The first step is to take the projection of *Y* onto *X*, $\hat {Y} = \mathbf {P}_{X} Y$, where **P**_*X*_ represents a projection matrix to the column space of *X*, denoted by *C*(*X*). Furthermore, we write *Y* as follows:

$$\begin{array}{*{20}l} Y = \mathbf{P}_{{X}}Y + (Y-\mathbf{P}_{{X}}Y) := \mathbf{P}_{{X}}Y + \mathbf{A}_{{X}}Y = \hat{Y} + \mathbf{A}_{{X}}Y, \end{array} $$

where **A**_*X*_=*I*−**P**_*X*_ represents a projection matrix to the space orthogonal to the column space of *X*. Accordingly, **A**_*X*_*Y* is orthogonal to *C*(*X*): for **v**∈*C*(*X*),

1 $$\begin{array}{*{20}l} \mathbf{v}^{\top} \mathbf{A}_{{X}}Y = 0. \end{array} $$

For underdetermined systems, *p*>*n*, as is common in spectral data, $\hat {Y}$ equals *Y*.

The second step is to decompose *X* into two orthogonal parts, one part that has the same range $\hat {Y}$ and another that is orthogonal to it:

$$\begin{array}{*{20}l} X = \mathbf{P}_{\hat{Y}}X + (X-\mathbf{P}_{\hat{Y}}X) = \mathbf{P}_{\hat{Y}}X + \mathbf{A}_{\hat{Y}}X. \end{array} $$

Thus, the space spanned by the columns of $\mathbf {A}_{\hat {Y}}X$, which is essentially a subspace in *X* and orthogonal to $\hat {Y}$, is a target of removal from *X* in the OSC procedure. The space $\mathbf {A}_{\hat {Y}}X$ is also orthogonal to *Y*: for $\mathbf {v} \in C(\mathbf {A}_{\hat {Y}}X)$,

2 $$\begin{array}{*{20}l} Y^{\top} \mathbf{v} = (\hat{Y} + \mathbf{A}_{{X}}Y)^{\top} \mathbf{v} = \hat{Y}^{\top} \mathbf{v} + (\mathbf{A}_{{X}}Y)^{\top} \mathbf{v} = 0, \end{array} $$

since $\hat {Y}$ is orthogonal to $\mathbf {A}_{\hat {Y}}X$ by definition and **v**, also in *C*(*X*), is orthogonal to **A**_*X*_*Y* by (1).

The third step is to find the first principal score vector **t**_*OSC*_ and the associated loading vector **p**_*OSC*_ from $\mathbf {A}_{\hat {Y}}X$, so that $\mathbf {t}_{OSC}\mathbf {p}^{\top }_{OSC}$ approximates to $\mathbf {A}_{\hat {Y}}X$. Let *X*^*ORTH*^ be the approximation of $\mathbf {A}_{\hat {Y}}X$ by $\mathbf {t}_{OSC}\mathbf {p}^{\top }_{OSC}$: $X^{ORTH}=\mathbf {t}_{OSC}\mathbf {p}^{\top }_{OSC}.$ The score vector **t**_*OSC*_, a direction or an OSC component, accounts for the maximum variance of $\mathbf {A}_{\hat {Y}}X$. Moreover, the score vector $\mathbf {t}_{OSC} \in \mathbf {A}_{\hat {Y}}X$ is orthogonal to $\hat {Y}$ by the definition of $\mathbf {A}_{\hat {Y}}X$ and also orthogonal to *Y* by (2):

3 $$\begin{array}{*{20}l} \mathbf{t}_{OSC}^{\top} Y = 0. \end{array} $$

Being in *C*(*X*), the score vector **t**_*OSC*_ is expressed as a linear combination of the columns of *X* with a weight vector **r**_*OSC*_:

4 $$\begin{array}{*{20}l} \begin{aligned} \mathbf{t}_{OSC} &= X \mathbf{r}_{OSC}, \text{~~or~~~} \\ \mathbf{r}_{OSC} &= X^{\dagger} \mathbf{t}_{OSC}, \end{aligned} \end{array} $$

where *X*^*†*^ is the Moore-Penrose pseudoinverse of *X*. Overall, the orthogonal-signal corrected *X* preserving predictive components, denoted by *X*^*OSC*^, writes:

5 $$\begin{array}{*{20}l} X^{OSC} := X - X^{ORTH} = X - \mathbf{t}_{OSC}\mathbf{p}^{\top}_{OSC} = X - X \mathbf{r}_{OSC} \mathbf{p}^{\top}_{OSC}. \end{array} $$

Here, weight vector **r**_*OSC*_ consists of weights of individual variables contributions to the construction of a space orthogonal to *Y*, which will be used to relate the input matrix *X* to the OPLSR vectors in the last step. Practically, it is necessary to limit the number of OSC components. To avoid over-fitting, which results in a loss of model generality, the number of OSC components should not be too high. Mostly, one or two OSC components are sufficient. The first OSC component often describes a base-line correction, and the second can serve as the correction of multiplicative effects [22]. In this study, we chose one OSC component in consideration of the intuitive interpretation and the sufficient amount of its variability in *X* in practice.

Now that *X*^*OSC*^ is found, we apply PLS to *X*^*OSC*^ and *Y*. The reason PLS is applied to *X*^*OSC*^ and not directly to *X* is that the covariance between *X* and *Y* equals that between *X*^*OSC*^ and *Y*:

$$\begin{array}{*{20}l} X^{\top} Y = \left(X^{OSC} + \mathbf{t}_{OSC}\mathbf{p}^{\top}_{OSC} \right)^{\top} Y = \left(X^{OSC}\right)^{\top} Y + \mathbf{p}_{OSC}\mathbf{t}_{OSC}^{\top} Y = \left(X^{OSC}\right)^{\top} Y, \end{array} $$

because **t**_*OSC*_ is orthogonal to *Y* by (3). Thus, the PLS finds a few score vectors of *X*^*OSC*^ that maximize the covariance between *X*^*OSC*^ score vectors and *Y* score vectors. The scores of *X*^*OSC*^,**t**=[ **t**_1_ **t**_2_ ⋯ **t**_*A*_ ] (of size *n*×*A*) corresponding to *A* predictive components, are not only orthogonal, but also obtainable by computing its associated weight vectors **r**=[ **r**_1_ **r**_2_ ⋯ **r**_*A*_ ] from the singular value decomposition of the covariance matrix (*X*^*OSC*^)^⊤^*Y*:

6 $$\begin{array}{*{20}l} \begin{aligned} \mathbf{t}_{j} &= X^{OSC} \mathbf{r}_{j}, \quad j=1,\cdots,A, \text{~~or} \\ \mathbf{t} &= X^{OSC} \mathbf{r}. \end{aligned} \end{array} $$

Accordingly, the corresponding loading of *X*^*OSC*^, denoted by *P*, is given by *P*=(*X*^*OSC*^)^⊤^**t**, meaning new weights of *X*^*OSC*^ are directly evaluated by **t**. Similarly, the loading of *Y*, denoted by *Q*, is computed by *Q*=*Y*^⊤^**t** and represents new weights of *Y* that lead to maximization of the covariance matrix using the *A* predictive components commonly applied to *X*^*OSC*^ and *Y* [23].

Overall, *X*^*OSC*^ and *Y* are decomposed into the following:

$$\begin{array}{*{20}l} X^{OSC} &= \mathbf{t} P^{\top} + E,\\ Y &= \mathbf{u} Q^{\top} + F, \end{array} $$

where **u** is the score matrix of *Y* given by **u**=*YQ*; and *E* and *F* are residual matrices.

By regressing *Y* on the score **t**, which is a compact representation of *X*^*OSC*^ based on the *A* predictive components, from the regression model *Y*∼**t***β*, the least-squares estimate of the coefficient *β* becomes $\hat {\beta } = (\mathbf {t}^{\top }\mathbf {t})^{-1}\mathbf {t}^{\top } Y = \mathbf {t}^{\top } Y$ due to the orthogonality of **t**. Then the prediction model $\hat {Y}$ is

7 $$\begin{array}{*{20}l} \hat{Y} = \mathbf{t} \hat{\beta} = \mathbf{t} \mathbf{t}^{\top} Y = X^{OSC} \mathbf{r} \mathbf{t}^{\top} Y = X^{OSC} \mathbf{r} Q^{\top} := X^{OSC} \hat{\beta}_{OPLSa}, \end{array} $$

where $\hat {\beta }_{OPLSa} = \mathbf {r} Q^{\top }$ is the regression vector using the orthogonal signal corrected *X*, *X*^*OSC*^. Furthermore, the prediction model $\hat {Y}$ can be rewritten as follows:

8 $$\begin{array}{*{20}l} \hat{Y} = X^{OSC} \hat{\beta}_{OPLSa} = \left(X - X \mathbf{r}_{OSC} \mathbf{p}_{OSC}^{\top}\right) \hat{\beta}_{OPLSa} := X \hat{\beta}_{OPLSb}, \end{array} $$

where $\hat {\beta }_{OPLSb}=\left (I-\mathbf {r}_{OSC} \mathbf {p}_{OSC}^{\top }\right) \hat {\beta }_{OPLSa}$ is the regression vector based on *X*. Both $\hat {\beta }_{OPLSa}$ and $\hat {\beta }_{OPLSb}$ consist of weights of individual variables *i*, *i*=1,⋯,*p*, contributing to the change of *Y*. In other words, a large absolute value of $\hat {\beta }_{OPLSa,i}$ or $\hat {\beta }_{OPLSb,i}$ indicates that the *i*th variable (*x*_*i*_) contributes substantially to the increase or decrease of *Y* depending on the sign. We notice that the use of $\hat {\beta }_{OPLSb,i}$ in contrast to $\hat {\beta }_{OPLSa,i}$ brings comprehensive examination of individual variable effects because it includes the presence of all *X* directions, both orthogonal and predictive. Thus we propose $\hat {\beta }_{OPLSb}$ as the final regression coefficient vector, regarding $\hat {\beta }_{OPLSa}$ as a side source for comparison. This comparison will be demonstrated in a simulation study and a real-life data example that follow. We will obtain the distributions of $\hat {\beta }_{OPLSa}$ and $\hat {\beta }_{OPLSb}$ to systemically select variables that significantly contribute to the change of *Y*.

### Permutation tests

Since the OPLS regression vectors, $\hat {\beta }_{OPLSa}$ and $\hat {\beta }_{OPLSb}$ are computed, the aim here is to test the significance of individual variables effects on the vectors. Let $\hat {\beta }_{OPLS}$ denote the two regression vectors generally. Permutation tests, a computer-based re-sampling method for achieving accuracy measures of statistical estimates from an approximating distribution [24], are widely used in the computation of variable importance and confidence intervals in random forests [25, 26].

The advantage of permutation tests is that it is straightforward to simulate empirical null distributions of complex statistics such as percentiles, odds ratios, or correlation coefficients. The exact distributions of OPLSR vectors are too complex to obtain, and so are even approximations to the distributions. Thus the permutation test is employed to test the significance of individual variables in the OPLSR vector.

To propose a permutation test procedure to test the significance of the $\hat {\beta }_{OPLS}$ coefficients, the basic procedures are presented as follows:

Step 1 Perform PLS with *Y* and *X*^*OSC*^.
Step 2 Obtain the observed values of $\hat {\beta }_{OPLS,i}$ for all *i*.
Step 3 For each variable *i*, repeat the following a large number of times (e.g., 999 times):
  Step 3.1 Randomize the values within the *i*th column of *X*^*OSC*^.
  Step 3.2 Perform PLS with *Y* and the permuted *X*^*OSC*^.
  Step 3.3 Obtain the realized value of $\hat {\beta }_{OPLS,i}$
Step 4 Estimate the *p*-value *p*_*i*_ for the *i*th variable (e.g., (the number of the realized equal to or larger than the observed in terms of absolute values+1)/1000).
Step 5 Choose a significance level *α*_*pm*_ so that variables in which *p*_*i*_<*α*_*pm*_ are selected.

For the randomization procedure at Step 3.1, one can choose several columns of *X* randomly, i.e., a portion of the total number of variables in one iteration to speed up the procedure since the total number of variables is usually quite large. We note that the proportion of 0.3 worked well in practice, producing the empirical null distributions of realized $\hat {\beta }_{OPLS,i}$ that were highly close to normality. In this manner, the realized values of $\hat {\beta }_{OPLS,i}$ can be regarded as emerging from a collection of the null hypotheses that the variables effects are insignificant. The motivation is that practically we can assume a substantial number of variables are negligible among the large number of variables, which is quite common in spectral data. The coefficients $\hat {\beta }_{OPLS,i}$ for the variable *i* become zero in theory if the variable *i* does not contribute to the construction of response variables *Y*. Accordingly, the *p*-value is computed according to the extreme ‘two-tailed’ directions at Step 4. We also note that at Step 4, the observed value is included as one of the possible values of the randomization procedure. To control the increase of Type I error due to multiple testing, a correction can be made at Step 5. For instance, one can adjust the significance level *α*_*pm*_ to be *α*_*pm*_/*p* using the Bonferroni procedure. In this study, we set *α*_*pm*_ to 0.05 due to its practicality. As a pre-processing step for the permutation test with significance level *α*_*f*_, we filter out the variables of which the individual correlation measures with *Y* are weak. This step eliminates unnecessary noisy variables that can act as contributing variables from the whole procedure, so that only filtered-in variables are considered. The level of *α*_*f*_ is considered as a tolerance level for the OPLSR method and usually set to 0.05. By changing the level of *α*_*f*_, we can control the number of variables included in the permutation test.

### Simulation and comparisons

**Fig. 1:**
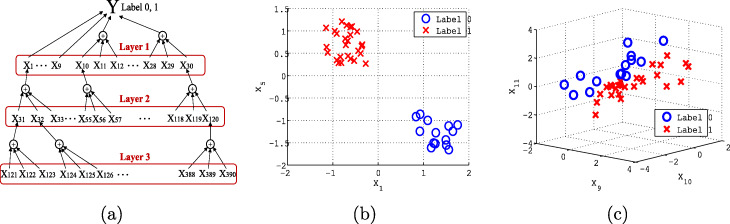
**a** A data model to generate 1000 variables has three layers of variable interation that contribute to the response *Y*. Layer 1 contains 30 variables, including eight strong variables (*x*_1_ to *x*_9_) and the other group-wise variables (*x*_10_ to *x*_30_). Each of the variables in Layer 1 is determined by three variables in Layer 2, and each variable in Layer 2 by three variables in Layer 3. The remaining 610 variables are randomly and independently assigned. **b** Two strong variables *x*_1_ and *x*_5_ separate the two labels of *Y*. **c** Three variables in Layer 1 jointly separate the two labels of response *Y*

To test the OPLS regression-based feature selection method, we performed a simulation study. We first generated a data matrix *X*(40×1000) and a response matrix *Y*(40×1), comprising 40 samples and 1000 variables per sample. Each element of *Y* was a Bernoulli random variable with success probability 0.4, so 16 elements of *Y* were set to 1 on average while the remaining 24 elements were set to 0. To generate 1000 variables, we used a three-layer network structure as shown in Fig. 1a.

In the first layer, the first 30 variables were generated to have high separation in *Y*: for *p*=1,…,4,*x*_*p*_∼*U*(0,1)+0.8−2*Y* and for *p*=5,…,9,*x*_*p*_∼*U*(0,1)−1.2−2*Y*, where *U*(*a*,*b*) is a random variable from a uniform distribution of range *a* and *b*. This means the variables from *x*_1_ to *x*_4_ individually had a high positive correlation with *Y* (labeled 0), whereas *x*_5_ to *x*_9_ were highly correlated with *Y* (labeled 1). For instance, Fig. 1b shows the plots of realizations of *x*_1_ and *x*_5_ and the aforementioned pattern that individually separate *Y*. The first 30 variables in the first layer represent strong individual variables that can identify pathological conditions. For *p*=10,13,…,28,

$$\begin{array}{*{20}l} \left[x_{p}~x_{p+1}~x_{p+2}\right]^{T} \sim N\left(0 + Y[1~2~3]^{T}, \left[ {\begin{array}{lll} 12 & 10 & 8 \\ 10 & 12 & 10 \\ 8 & 10 & 12 \end{array}} \right] \right), \end{array} $$

where *N*(*μ*,*Σ*) represents a multivariate normal distribution with mean *μ* and covariance *Σ*. Figure 1c shows plots of realizations of three variables in the layer, generated as above, jointly discriminate *Y*. The variables *x*_*i*_,*i*=10,…,30, in the first layer represent strong group-wise variables that strongly discriminate pathological conditions.

The next 90 variables from *x*_31_ to *x*_120_ in the second layer and the next 270 variables from *x*_121_ to *x*_390_ in the third layer were generated so that those variables contribute to the overall response *Y* in a composite and aggregate manner. For instance, *x*_1_ in the first layer is clearly separable by a combination of *x*_31_,*x*_32_, and *x*_33_ in the second layer. Specifically, the generation of three variables is based on the value *x*_1_ so that the sum of the three will be close to *x*_1_ as follows: we set $x_{31} = \frac {u_{1}}{u_{1} + u_{2} +u_{3}}(x_{1} + \epsilon), x_{32} = \frac {u_{2}}{u_{1} + u_{2} +u_{3}}(x_{1} + \epsilon)$, and $x_{33} = \frac {u_{3}}{u_{1} + u_{2} +u_{3}}(x_{1} + \epsilon)$ using independently generated *u*_1_,*u*_2_, and *u*_3_ from *U*(0,1) and *ε* from *N*(0,*x*_1_/10). We notice that *x*_31_+*x*_32_+*x*_33_=*x*_1_+*ε*. The remaining 610 variables from *x*_391_ to *x*_1000_, comprising a noise layer, were randomly and independently generated from *N*(0,1) so as to have a weak correlation with *Y*. They represent the presence of inherent noise. The adopted three-layers structure is a simulated example of multiple layers of spectral collinearity, interaction, and regulation in complex biological systems. For instance, a biological system for nutritional metabolomics reflects such a layered structure with linked transports [27].

Using the simulated data, we tested the performance of the proposed methods in comparison with the false discovery rate (FDR) method to detect the known variables in the three layers. Focusing on the effects of individual variables and controlling family-wise Type I error, FDR serves as a baseline method to compare the OPLSR method with. The two types of OPLS regression vector, $\hat {\beta }_{OPLSa}$ and $\hat {\beta }_{OPLSb}$, were considered. The number of predictive components for the OPLS regression model, *A* as in (6), varied from 1 to 3 so as to determine its effect upon the performance. The filtering level *α*_*f*_ for the OPLSR method and the *q*-value for FDR varied from 0.01 to 0.05 and 0.10. We repeated this test 1000 times, and in each repetition for each method, the number of variables that were found within the three layers were counted. No variables among the random 610 variables were found for each of the two methods.

**Fig. 2:**
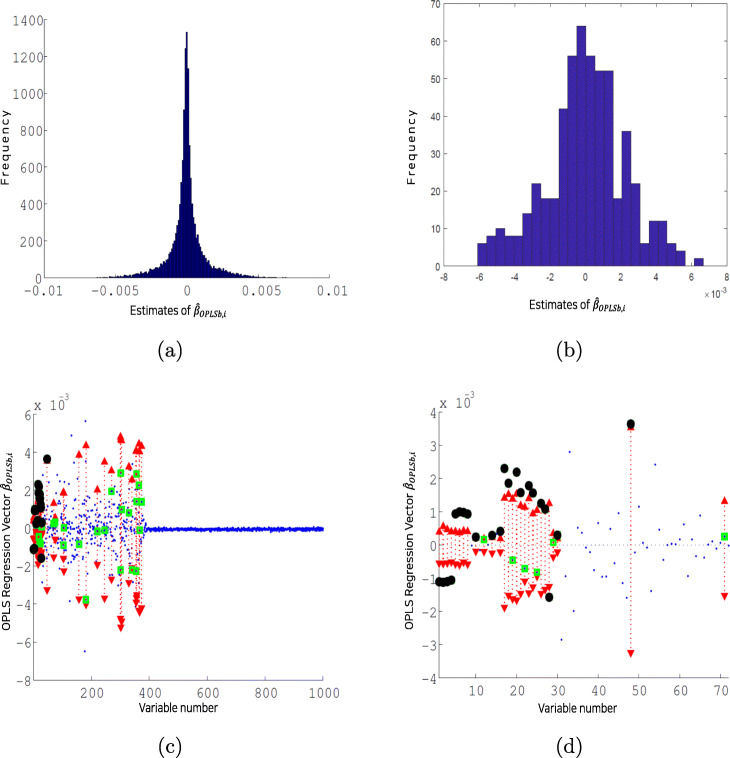
**a** Illustration of the empirical null distribution of realized $\hat {\beta }_{OPLSb,i}$, which is close to normality, is shown. **b** Illustration of the empirical null distribution of realized $\hat {\beta }_{OPLSb}$ for the variable with the largest confidence interval is shown. **c** Illustration of the regression vector $\hat {\beta }_{OPLSb}$ is shown. The blue dots represent observed $\hat {\beta }_{OPLSb,i}$, the green squares represent filtered-in variables according to the significance level *α*_*f*_, the red arrows with dotted lines represent achieved 1−*α*_*pm*_ confidence intervals, and the black circles represent selected significant variables. **d** Zoomed-in version of (**c**) for the variables from 1 to 72 with 22 significant variables selected. The filtered-in variable 71 (*x*_71_), for example, was not selected since the observed $\hat {\beta }_{OPLSb,i}$ is within the confidence interval of realized $\hat {\beta }_{OPLSb,i}$

To illustrate the performance of the proposed method, Fig. 2 shows illustrations of the empirical null distribution of the realized $\hat {\beta }_{OPLSb,i}$ and regression vector $\hat {\beta }_{OPLSb}$. The empirical null distribution of $\hat {\beta }_{OPLSb,i}$ in Fig. 2a, obtained by the permutation procedure, is closely normally distributed and provides a basis for testing the observed $\hat {\beta }_{OPLSb,i}$. Figure 2b also shows the empirical null distribution of the realized $\hat {\beta }_{OPLSb}$ for the variable with the largest confidence interval. This illustration, being the worst case, demonstrates that the distribution of the realized regression coefficients for individual variables can be sufficiently approximated to a normal distribution by the setting. The regression vector $\hat {\beta }_{OPLSb}$ in Fig. 2c highlights the filtered-in variables according to the *α*_*f*_-level filtering (green squares), confidence intervals of the permutation procedure (red arrows with dotted lines), and selected variables (black circles) along with the observed regression vector $\hat {\beta }_{OPLSb,i}$ (blue dots). We notice that observed $\hat {\beta }_{OPLSb,i}$ for *i*=1,⋯,390 are substantially larger than those for the rest, which indicates the regression vector reflects the network structure for the data set. The selected variables gather in the first layer as shown in Fig. 2d, which implies the testing procedure of the method suitably separate known important variables. Particularly, we notice that the variables (from *x*_1_ to *x*_8_) are positioned accordingly with their contributions to the labels. For example, the realized value $\hat {\beta }_{OPLSb,i}$ for *x*_1_ is negative, implying that the increase of *x*_1_ results in the increase of label 0 instead of label 1. This is in accordance with the behavior of *x*_1_ in Fig. 1b. In Fig. 2d we also observe that the filtered-in variable 71 (*x*_71_), for example, was not selected since the observed $\hat {\beta }_{OPLSb,i}$ is within the confidence interval of the realized $\hat {\beta }_{OPLSb,i}$.

Additionally, Table 1 shows the amounts of variation of *X*^*ORTH*^,*X*^*OSC*^, and *Y* in the OPLSR method. The first OSC component and the first PLS component accounted for more than 96% of the total *X* variation and 99% of the total *X*^*OSC*^, respectively. It is an empirical evidence that using the first OSC component and the first one or two PLS components is sufficient. The first PLS component accounted for more than 99% of the *Y* variation in the simulation study. In fact, we used the first OSC component when correcting *X* in (5). Table 2 shows the average numbers of selected variables for OPLSR and FDR according to significance level *α*. Since the variables in layer 1 are significant, the selected variables in layer 1 mean the recall rate. The OPLSR method is divided into $\hat {\beta }_{OPLSa}$ (denoted by OPLSR _*a*_) and $\hat {\beta }_{OPLSb}$ (denoted by OPLSR _*b*_). We chose significance level *α* as *q*-value for FDR and *α*_*f*_ for OPLSR. We note that the levels for the methods are not strictly equivalent by themselves, yet we compare them in that they practically adjust the number of selected variables.

**Table 1:** Amounts of variation of *X*^*ORTH*^,*X*^*OSC*^, and *Y* in the OPLSR method for the simulation study are shown. The first OSC component and the first PLS component accounted for more than 96% of the total *X* variation and 99% of the total *X*^*OSC*^, respectively. The first PLS component accounted for more than 99% of the *Y* variation in the simulation study

			PLS						PLS		
	OSC		1	2	3		OSC		1	2	3
*X*^*ORTH*^	1	0.967					2	0.967			
*X*^*OSC*^		0.033	0.991	0.002	0.002			0.033	0.994	0.002	0.001
*Y*		1.00	1.00	0.000	0.000			1.00	1.00	0.000	0.000

**Table 2:** The average numbers of selected variables in the layers for each method of OPLSR, according to $\hat {\beta }_{OPLSa}$ (denoted by OPLSR _*a*_) and $\hat {\beta }_{OPLSb}$ (denoted by OPLSR _*b*_), and FDR; and significance level *α*, meaning *α*_*f*_ for OPLSR _*b*_ and *q*-value for FDR, are shown. This shows that OPLSR _*a*_ found more variables consistently while OPLSR _*b*_ worked comparably to FDR

*α*		0.01				0.05				0.10		
		Layer				Layer				Layer		
		1	2	3		1	2	3		1	2	3
OPLSR _*a*_		24.4	0.837	0.580		25.0	3.79	5.61		25.1	5.32	10.0
OPLSR _*b*_		18.1	0.190	0.137		20.8	0.987	1.24		22.9	2.85	3.68
FDR		21.3	0.020	0.000		22.1	0.100	0.027		22.8	0.103	0.010

The results for this simulation study show that OPLSR _*a*_ consistently found more variables than FDR. The performance of OPLSR _*b*_, working quite well, was comparable with that of FDR while surpassing FDR for big *α*.

## Experiments

**Fig. 3:**
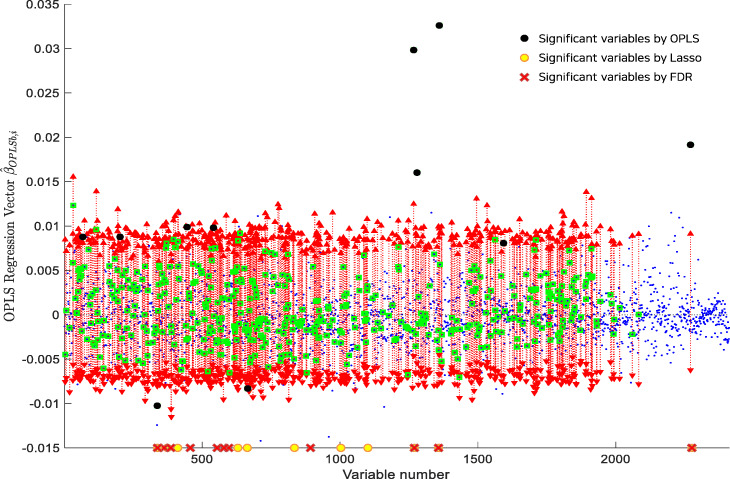
The empirical null distribution of the realized $\hat {\beta }_{OPLSb,i}$ for the mass spectrometry data is shown with two-sided 95% confidence intervals by red rectangles. The black dots mean significant m/z variables by the permutation testing of OPLS regression. The yellow dots and the red crosses illustrate siginificant variables by the Lasso and the FDR, respectively

We applied the proposed method in the examination of a high-resolution metabolomics data set. We show the use of the proposed method, and furthermore analysis will follow with the next dataset. The code and data are uploaded to the following GitHub url: https://github.com/leegs52/OPLSR. The metabolomics data set consists of 127 samples screened for bile acids by a BioQuant colormetric kit [28]: 64 samples have bile acids present and the others have bile acids absent. The metabolomic profiling was performed using liquid chromatography (LC) coupled to high-resolution mass spectrometry by an Orbitrap FTQ-Velos mass spectrometer. Peak extraction and quantification of ion intensities were performed by an adaptive processing of liquid chromatography mass spectroscopy software package that produced 7068 m/z (mass divided by charge number of ions) values. We aimed to extract significant metabolites, for example the top 1*%*, that separate bile acid present and bile acid absent. Application of the OPLSR method to the data matrix *X* of size 127×7068 and response *Y* of size 127×1 yielded the empirical null distribution of OPLS regression vector $\hat {\beta }_{OPLSb}$. Figure 3 shows two-sided 95% confidence intervals (red rectangles) and significant m/z variables (black circles) using the permutation testing of OPLS regression. The small blue dots represent the OPLS regression coefficients. The proposed method was able to filter out significant variables in that the number of found significant variables (black circles) by the OPLS is 11 while the number of variables (green circles) by individual coefficient testing of regression analysis is 461. The number of significant variables by either Lasso (marked by yellow circles) or FDR (marked by red crosses) are 11, like the OPLS. For information, all three of them found *x*_1267_,*x*_1360_, and *x*_2271_ the significant variables.

**Fig. 4:**
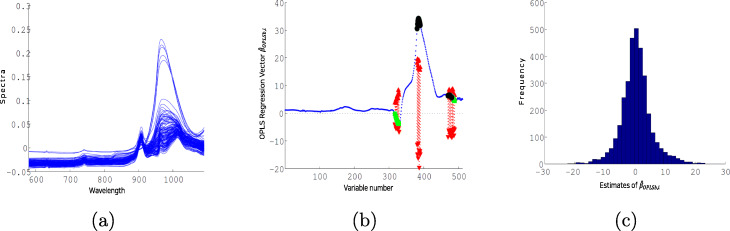
(**a**) Overall 110 NIR spectra for temperatures of 30, 40, 50, 60, and 70 ^∘^C are shown. Illustration of (**b**) the regression vector $\hat {\beta }_{OPLSb}$ and (**c**) the empirical null distribution of the realized $\hat {\beta }_{OPLSb,i}$ are shown for the NIR spectra. The wavelength ranges of the selected variables were from 960 to 970 nm (*x*_381_ to *x*_391_) and from 1049 to 1060 nm (*x*_470_ to *x*_481_)

We also applied the proposed method to a near-infrared (NIR) spectroscopic technique, which is a useful tool for chemical process analysis in research such as pharmaceutical, medical diagnostics, and agrochemical quality. Measured NIR spectroscopic spectra are influenced by external process variables such as temperature, pressure, and viscosity. The difficulty in keeping these variables unchanging and the necessity to change their value during the process (e.g., setting temperature and pressure in batch processes) make it necessary to assess the influence on the NIR spectra. Wülfert et al. took short-wave NIR spectra of ethanol, water, and 2-propanol mixtures at different temperatures to assess the influence of those temperature-induced spectra variations [29]. The proposed method was applied to the 22 spectra of the mixtures at each temperature of 30, 40, 50, 60, and 70 ^∘^C (*n*=22×5=110). The used wavelengths are from 580 to 1091 nm, resulting 512 variables (*p*=512). The overall 110 spectra are shown in Fig. 4a.

The data set consists of the 110 spectra as *X* (110×512) and temperatures as *Y* (110×1). For the purpose of testing predictive power, the data set was randomly split into a training set with 70% of the whole for building a prediction model and a test set with the remaining 30% for estimating the predictive quality of that model. Using the selected variables for each method of OPLSR _*a*_, OPLSR _*b*_, FDR, and Lasso, we carried out regression analysis to predict *Y* of the test set and calculated the mean-squared error (MSE) of prediction and the respective amount of *Y* variance being described by the model, *Q*^2^, as follows:

$$\begin{array}{*{20}l} Q^{2} = 1 - \frac{\sum_{i}(Y_{i,test} - \hat{Y}_{i,test})^{2}}{\sum_{i} Y_{i,test}^{2} }. \end{array} $$

This was repeated 3,000 times. We also measured the precision, denoted by P, and the number of selected variables, denoted by N, as additional information on the performance of each method. The precision P is the fraction of correctly selected variables compared to all selected variables. For the NIR spectra of temperatures, Fig. 4a shows that the variation between them is highly significant around 980 nm with a peak maximum [30]. By considering each wavelength within the 930-1060 nm as the ground-truth features, we verified the selected variables.

The illustrations of the proposed method for the NIR spectra are shown in Fig. 4b and c, which depict the regression vector $\hat {\beta }_{OPLSb}$ and the empirical null distribution of realized $\hat {\beta }_{OPLSb,i}$, respectively. The wavelength ranges of the selected variables were from 960 to 970 nm (*x*_381_ to *x*_391_), which corresponds to the free OH 2nd overtone, and from 1049 to 1060 nm (*x*_470_ to *x*_481_), which belongs to the hydrogen-bonded OH groups [31]. This finding is consistent with the chemical observation that temperature effects are related to the absorbance of molecule overtones and the cluster size of hydrogen-bonded molecules [29]. The empirical null distribution in Fig. 4c, following closely normality, supply the proposed method with the basis of the employed permutation test.

Table 3 shows the comparison results in terms of evaluation metrics and the number of selected variables. We observed that the OPLSR method reliably finds significant variables among the spectra most of the iterations since its precision outperformed FDR and Lasso. It is not surprising because the proposed method examines the effects of variables in a collective manner by considering the covariance structure and the effects of both orthogonal and predictive components at the same time. The regression performance for the variables selected by Lasso achieved relatively high predictive performance. Though Lasso was unsatisfactory in the precision. For all criteria of the precision, MSE, and *Q*^2^, OPLSR _*b*_ outperformed FDR consistently. The approach OPLSR _*a*_ also outperformed FDR consistently. The method OPLSR _*b*_ outperformed OPLSR _*a*_ for *α*_*f*_=0.01,0.05 in terms of the precision, MSE, and *Q*^2^, while there was little difference between the two for *α*_*f*_=0.10.

**Table 3:** Performance of each method of OPLSR _*a*_, OPLSR _*b*_, FDR, and Lasso is shown with respect to each parameter (*q*-value for FDR, *λ* for Lasso, and *α*_*f*_ for the other two). The precision and the number of selected variables were denoted by P and N, respectively. The boldfaced numbers indicate the ones which outperformed the others

Method		MSE	*Q*^2^	P	N
*OPLSR*_*a*_	*α*_*f*_=0.01	12,332	0.86	0.871	11.0
	*α*_*f*_=0.05	8,989	0.89	0.701	16.2
	*α*_*f*_=0.10	**6,829**	0.91	0.640	22.7
*OPLSR*_*b*_	*α*_*f*_=0.01	10,896	0.88	**0.915**	11.7
	*α*_*f*_=0.05	8,704	0.91	0.860	28.3
	*α*_*f*_=0.10	7,081	**0.92**	0.818	41.2
*FDR*	*q*=0.01	33,038	0.64	0.750	9.0
	*q*=0.05	12,123	0.86	0.714	36.6
	*q*=0.10	11,194	0.85	0.536	40.1
*Lasso*	*λ*=0.01	7,120	0.91	0.545	6.6
	*λ*=0.10	10,796	0.89	0.477	4.0
	*λ*=0.50	14,028	0.81	0.496	2.2

Additionally, in order to demonstrate the robustness of the proposed method, we conducted down-sampling and then performed another experiment under the same conditions of Table 3. The down-sampled data set was randomly drawn from NIR spectra data (*n*=110), and it contains 80 samples, i.e., about 70 percent of total. For the down-sampled data, Table 4 presents the performance comparison for the four methods, demonstrating the degree of robustness.

**Table 4:** Performance of each method on downsampled data. The boldfaced numbers indicate the ones which outperformed the others

Method		MSE	*Q*^2^	P	N
*OPLSR*_*a*_	*α*_*f*_=0.01	10,972	0.83	0.883	8.90
	*α*_*f*_=0.05	9,105	0.87	0.777	24.4
	*α*_*f*_=0.10	**5,709**	0.91	0.658	22.8
*OPLSR*_*b*_	*α*_*f*_=0.01	10,133	0.85	**0.884**	10.4
	*α*_*f*_=0.05	8,811	0.89	0.881	31.0
	*α*_*f*_=0.10	7,782	**0.93**	0.848	48.2
*FDR*	*q*=0.01	23,116	0.65	0.701	6.0
	*q*=0.05	10,330	0.85	0.685	39.5
	*q*=0.10	13,194	0.85	0.554	40.0
*Lasso*	*λ*=0.01	6,235	0.90	0.502	6.3
	*λ*=0.10	9,886	0.88	0.494	3.2
	*λ*=0.50	13,124	0.80	0.498	2.1

## Conclusions

We presented a feature selection method based on orthogonal-signal corrected PLS regression vectors to identify significant variables associated with the response characteristics. The proposed OPLSR method integrates PLS with orthogonal signal correction and permutation tests. To remove unnecessary variation in the input variables and improve interpretability of PLS regression vectors, orthogonal signal correction was applied first. The orthogonal-signal corrected PLS procedure reflects the variable interrelationships under complex systems, which are easily represented in a network structure. The two types of regression vectors from the model, carrying information on variables contribution to response characteristics, were derived and investigated in both a simulation study and a real-life spectra study in contrast to FDR and Lasso. To select the significant variables from the regression vectors, we applied a permutation test that generates empirical null distributions of variable effects on the response characteristics. The adopted permutation test was provided with the filtering rate, a pre-defined tolerance level for the whole selection procedure for eliminating unnecessary noisy variables, and was implemented efficiently by taking advantage of a collection of insignificant variables. Through simulations, we observed that the proposed method well captured the predefined network structures and successfully found the known variables. We demonstrated this method with real-life metabolomics and NIR spectra data, the finding variables that achieve a good level of predictive power and accurately relate to the chemical observations. For future research, we hope to investigate the effect of imbalance classes in the feature selection.

## Data Availability

The implemented MATLAB package is available from the following url address: https://github.com/leegs52/OPLSR
